# Exosomes in Parkinson: Revisiting Their Pathologic Role and Potential Applications

**DOI:** 10.3390/ph15010076

**Published:** 2022-01-07

**Authors:** Yassamine Ouerdane, Mohamed Y. Hassaballah, Abdalrazeq Nagah, Tarek M. Ibrahim, Hosny A. H. Mohamed, Areej El-Baz, Mohamed S. Attia

**Affiliations:** 1 Faculty of Medicine, Saad Dahlab University, Blida 09068, Algeria; ouerdaneyassamine@gmail.com; 2 Faculty of Pharmacy, Zagazig University, Zagazig 44519, Egypt; 14129@stemegypt.edu.eg (M.Y.H.); Abdalrazeqnagah@gmail.com (A.N.); hosnyahmedhosny2020@gmail.com (H.A.H.M.); a.elbaz21@pharmacy.zu.edu.eg (A.E.-B.); 3 Department of Pharmaceutics, Faculty of Pharmacy, Zagazig University, Zagazig 44519, Egypt; telmetwally@zu.edu.eg

**Keywords:** exosomes, neurodegenerative diseases, α-synuclein, Parkinson’s disease, therapeutic, biomarker, lewy bodies

## Abstract

Parkinson’s disease (PD) is a progressive neurodegenerative disorder characterized by bradykinesia, rigidity, and tremor. Considerable progress has been made to understand the exact mechanism leading to this disease. Most of what is known comes from the evidence of PD brains’ autopsies showing a deposition of Lewy bodies—containing a protein called α-synuclein (α-syn)—as the pathological determinant of PD. α-syn predisposes neurons to neurotoxicity and cell death, while the other associated mechanisms are mitochondrial dysfunction and oxidative stress, which are underlying precursors to the death of dopaminergic neurons at the substantia nigra pars compacta leading to disease progression. Several mechanisms have been proposed to unravel the pathological cascade of these diseases; most of them share a particular similarity: cell-to-cell communication through exosomes (EXOs). EXOs are intracellular membrane-based vesicles with diverse compositions involved in biological and pathological processes, which their secretion is driven by the NLR family pyrin domain-containing three proteins (NLRP3) inflammasome. Toxic biological fibrils are transferred to recipient cells, and the disposal of damaged organelles through generating mitochondrial-derived vesicles are suggested mechanisms for developing PD. EXOs carry various biomarkers; thus, they are promising to diagnose different neurological disorders, including neurodegenerative diseases (NDDs). As nanovesicles, the applications of EXOs are not only restricted as diagnostics but also expanded to treat NDDs as therapeutic carriers and nano-scavengers. Herein, the aim is to highlight the potential incrimination of EXOs in the pathological cascade and progression of PD and their role as biomarkers and therapeutic carriers for diagnosing and treating this neuro-debilitating disorder.

## 1. Introduction

Neurodegenerative diseases (NDDs) are considered irreversible deterioration and loss of neurons in different areas of the central nervous system (CNS). Therefore, neuronal damage occurring in the intellectual or cognitive locus can cause significant worsening in the patient’s clinical condition and lifestyle. Recently, it has been acknowledged that genetic and environmental factors can invoke certain types of NDDs [1].

Over the past years, NDDs prognosis was problematic because of poor drug access to the brain. Thus, the systemic delivery of drugs to the CNS is still challenging. Various factors may impede the drug uptake, such as extensive first-pass metabolism, reduced elimination half-life (tt2), and the possible side effects that may occur when reaching the non-target peripheral tissues [2]. The treatment approach for Parkinson’s disease (PD) is to compensate for the dopamine (DA) depletion in the brain and improve the motor symptoms. The first DA analog is levodopa (L-Dopa). Patients treated with this agent have experienced a functional benefit of decreased motor and non-motor symptoms [3].

PD is one of the most common NDDs, and its incidence is proportional to age. It emerged as an age-related disease since it exhibits a high prevalence of 0.41 per 100,000 in individuals over 40 years and 1900 per 100,000 in individuals over 80 years [4]. In addition, the disease’s pathogenicity is due to the degeneration of dopaminergic neurons at the substantia nigra pars compacta, also named as the dark substance, which is the DA-producing region. This region can control body movement, cognitive functions, and emotional activities.

Hence, most signs of PD are related to motor disability, such as bradykinesia, tremors, and rigidity [4,5]. These signs appear at one side of the body, worsen, and affect the other side. Some cognitive disorders such as dementia, sleep disturbance, anxiety, depression, psychosis, and speech disorders may appear [6]. Furthermore, Lewy bodies accumulation is the pathological determinant of PD. They are spherical and contain a protein called α-synuclein (α-syn). These bodies appear after the depletion of dopaminergic neurons at the substantia nigra pars compacta [7,8], explaining why α-syn gene overexpression or mutation is strongly associated with PD and dementia with Lewy bodies accumulation [9,10].

Furthermore, genes have a vital role in the progression of PD; for example, a mutation in the leucine-rich repeat kinase 2 (LRRK-2) gene can lead to familial or classical PD as it contributes to Lewy bodies aggregation [11,12].

Exosomes (EXOs) are intracellular membrane-based vesicles with diverse compositions involved in biological and pathological processes [13]. Until now, EXOs’ function in the brain is not fully understood. Still, it is found that these vesicles can regulate cellular communication within the CNS and perform an essential role in keeping the brain’s physiology. EXOs can mediate the myelination and axons survival by controlling the neuron-oligodendrocytes communication, as EXOs release is stimulated by glutamate secretion and then uptaken by neurons through endocytosis [14,15,16]. Moreover, EXOs have a neuroprotective role, which is observed upon the addition of EXOs derived from oligodendrocytes to cultured neurons leading to an increment in cell viability even under stress conditions [14,15]. It was reported that EXOs had a synaptic plasticity role as the microtubule-associated protein 1b (MAP1b), a synaptic plasticity-associated protein, is released from EXOs during neurons depolarization [14].

EXOs are considered ideal non- or less invasive biomarkers for diagnosing various diseases, such as PD, as they can be detected in different body fluids and tissues [17]. Furthermore, different proteins from host cells enriched in EXOs can be used as biomarkers themselves [18]. The potentiality of PD diagnosis using EXOs arises from the accumulation of α-syn, a pivotal PD manifestation transmitted among neurons through EXOs. EXOs release is stimulated by NLR family pyrin domain-containing three protein (NLRP3) inflammasome activation in microglia and subsequent release of pro-inflammatory cytokines, which are significant events related to PD progression [19].

The potential of EXOs for drug delivery is based on their size and competence to transfer biological materials to recipient cells [20]. Naturally produced EXOs have evolved to surpass the challenges for other drug delivery systems, including liposomes and polymeric nanoparticles [21,22]. However, the high stability and circulation capability of liposomal systems are limited by the increased toxicity and immunogenicity [23]. Though polymeric nanoparticles can solve the stability issue, their toxicity and biocompatibility remain a major concern, especially when using non-biodegradable polymers [24]. Owing to their natural origin, EXOs can exhibit a limited long-term accumulation in most organs, leading to almost no systemic toxicity [25]. EXOs are highly biocompatible with various biological systems; thus, they are selected as natural drug delivery vehicles for various therapeutic cargo [26]. Therefore, an exosomal drug delivery system with minimal toxicity, high biocompatibility, tissue and tumor targeting, and long-circulating tt2 becomes a more practical choice, conquering the drawbacks of liposomes or polymeric nanoparticles [21]. 

## 2. Overview of Extracellular Vesicles

Over the past decade, the small endosomal derived membrane microvesicles, EXOs, have gained more attention and interest. In addition to their presence in the extracellular space, they are secreted from cells as a cellular waste of cell damage or by-products of cell homeostasis without remarkable effect on neighboring cells [27]. Those functional extracellular vesicles can carry a complex cargo of proteins, lipids, and nucleic acids and deliver these cargoes to the target cells [28]. Since EXOs can be released by all eukaryotic cells, their cargos are considerably different, corresponding to the function of originated cells and their current state. Therefore, EXOs and their cargoes can indicate various diseases such as neurodegenerative, cardiovascular, renal, and metabolic diseases [29].

### 2.1. EXOs Biogenesis

EXOs are generated from late endosomes by inward budding of the limited multivesicular body (MVB) membrane and is followed by the invagination of late endosomal membranes and the formation of intraluminal vesicles (ILVs) within large MVBs. During this process, specific proteins are incorporated into the invaginating membrane while the cytosolic components are engulfed within the ILVs. Most ILVs, referred to as “EXOs”, are released into the extracellular space upon fusion with the plasma membrane [30].

### 2.2. EXOs Structure

Various elements have been identified in EXOs in terms of specially sorted proteins, lipids, nucleic acids representing the structure complexity and potential functional diversity of EXOs [31]. EXOs are highly enriched in multifunctional proteins, for example, tetraspanins, heat shock proteins, and MVB formation proteins. These proteins participate in cell penetration, antigen binding, and release of EXOs, respectively [32].

**Figure 1:**
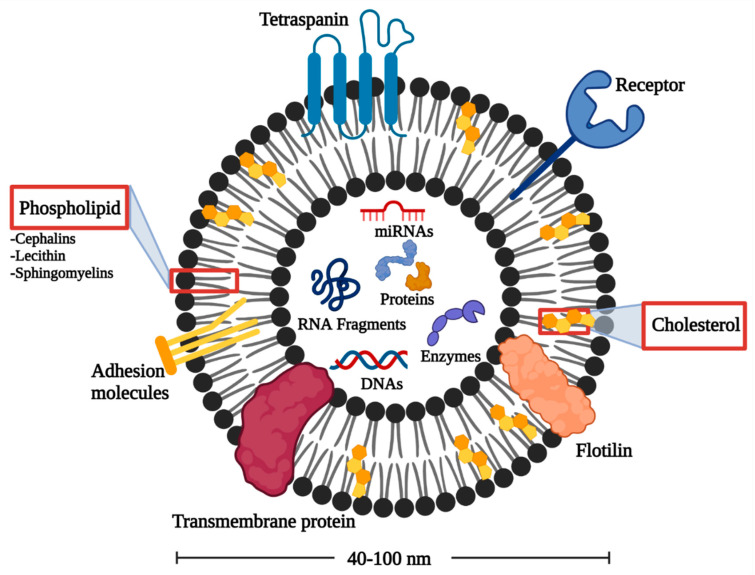
Architecture of EXOs.

Furthermore, the lipid architecture displays the EXOs bioactivity, as shown in Figure 1. EXOs are enriched in cholesterol, phosphatidylserine, sphingomyelin, phosphatidic acid, prostaglandins, and leukotrienes. These lipid components support the structural rigidity and stability of EXOs [33]. In addition, EXOs possess some lipolytic enzymes capable of producing various bioactive lipids to be internalized into target cells with a concentration of lipid mediators within the endosomes. Therefore, EXOs can offer diagnostic and prognostic information about various lipid-related diseases [27].

EXOs also contain various RNAs that can be incorporated into recipient cells. MicroRNAs (miRNAs) are abundant in human plasma-derived exosomal RNA species [34]. When miRNAs are packed into EXOs, they show unidirectional transfer between cells with the formation of an intercellular trafficking network that can produce phenotypic changes in recipient cells [35]. Besides miRNAs, long non-coding RNAs (lncRNAs) and circular RNAs (circRNAs) are present in EXOs influencing several biological processes such as the development of tumor and regulation of tumor microenvironment [36]. In addition, CircRNAs are proposed as a possible tumor diagnostic marker owing to their high stability and non-susceptibility to exonuclease cleavage [37].

### 2.3. Isolation of EXOs

Several methods have been used for EXOs isolation from the natural biofluids such as blood, urine, and cerebrospinal fluid (CSF). The most used technique is differential ultracentrifugation, which separates the needed particles according to their size and density; this technique is considered the gold standard for exosomal isolation [38]. Lately, classical techniques have waned due to modern technological advancement; thus, other methods have emerged; the disadvantage that made researchers revert to the differential ultracentrifugation technique is the possible contamination of the final product with same-sized particles creating exosomal aggregates [39]. Precipitating agents such as polyethylene glycol-based precipitation, immunoaffinity capture, microfluidics, and size-exclusion chromatography have emerged as alternatives, and each technique has specific criteria and strength points [40]. It is believed that using cost-effective approaches that are readily available will make EXOs applicable in treating NDDs in the foreseeable future.

## 3. Pathogenesis of PD

### 3.1. The α-Syn Role

Misfolding and aggregation of α-syn and its deposition in Lewy bodies result in autonomous-mediated neurotoxicity linked to PD. The pathogenesis of PD is mainly via the non-cell autonomous-mediated neurotoxicity and cell-to-cell transmission of α-syn [41,42].

**Figure 2:**
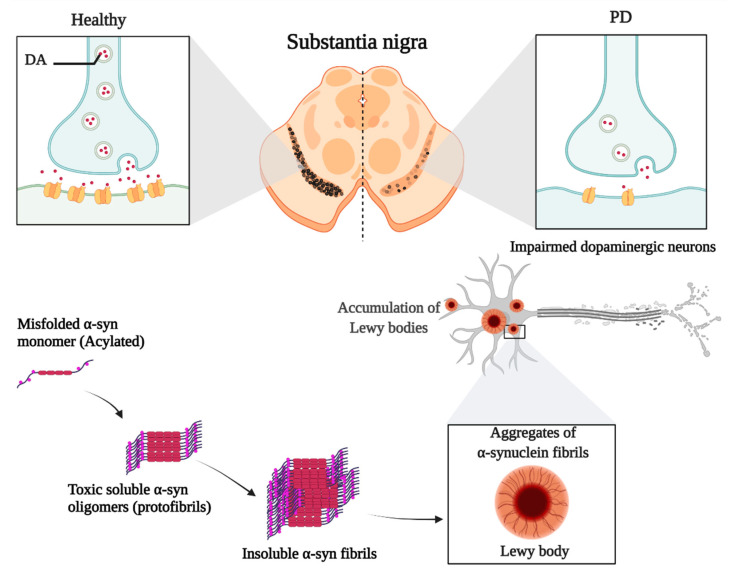
The role of α-syn in the development of PD.

α-syn monomers are amino-terminally acylated and thereby adopt a compact conformation. They can assemble into oligomers, and the latter continue to aggregate, forming soluble protofibrils or filaments; if a change in structure occurs, these filaments become insoluble fibrils [43,44]. Noting that the prefibrillar oligomers are more toxic than mature aggregated fibrils due to their ability to seed and accelerate the aggregation of α-syn monomers (Figure 2). Several reasons made it essential to focus on the widespread of these aggregates in PD. First, the larger oligomers or protofibrils have been shown to impair the function of several cytosolic organelles such as the mitochondria and endoplasmic reticulum, thereby disrupting the electrophysiological function of the synapse. Second, consider the potential spreading of α-syn aggregates between interconnected brain regions acting as seeds to propagate disease.

### 3.2. The Role of EXOs in Spreading α-Syn

**Figure 3:**
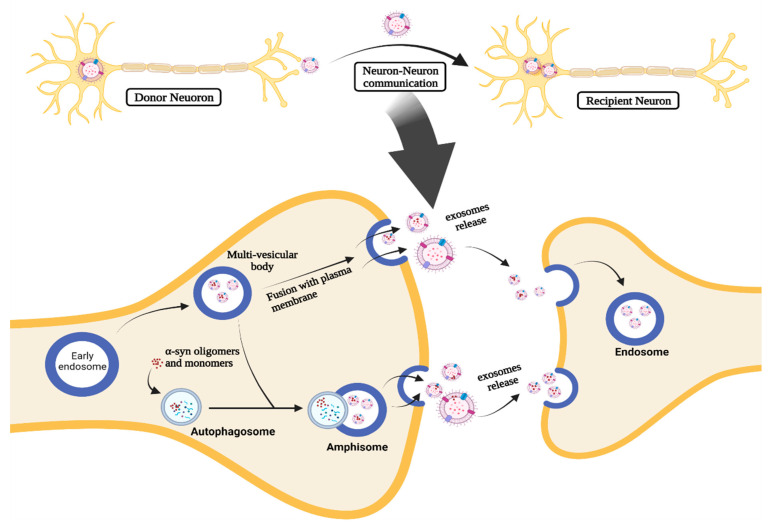
The role of EXOs in cell-to-cell communication in PD.

EXOs are molecular-carrying vesicles that have an essential role in transferring molecules, including microRNAs (miRNAs) and proteins, between neighboring cells as a part of cell-to-cell communication [19,45]. Thus, EXOs are essential components in different disease pathogenic mechanisms via cell-to-cell communication, which is the basis of their role in PD pathogenesis and progression. They may promote the PD progression by regulation, uptake, and transfer of aberrant α-syn and inflammatory mediators, transferring them to neighboring cells [19,41,45]. It has been reported that EXOs can shuttle α-syn oligomers from damaged neurons to normal neurons (Figure 3), leading to induction of aggregates formation and cell death [46]. In addition, EXOs can increase the likelihood of neurotoxicity mediated by α-syn since these oligomers associated with EXOs possess higher cellular uptake and neurotoxicity than non-extracellular vesicles (EVs) oligomers [41,46,47,48].

The α-syn-associated EXOs can be released through the intracellular vesicle trafficking process (Figure 3). When the early endosome process takes place, α-syn is internalized into ILVs and accumulates inside the MVB. Afterward, MVB containing ILVs undergoes fusion with the plasma membrane and releases ILVs as EXOs [41,49,50]. MVBs can also fuse with autophagosomes forming amphisomes and autophagic intermediates that can fuse with the plasma membrane to release EXOs containing α-syn [41,42].

Several in vitro studies demonstrated that EXOs could accelerate the aggregation of α-syn when EXOs were isolated from CSF of PD patients [41,46]. In contrast, EXOs isolated from the CSF of control patients did not influence the α-syn aggregation in any manner [1,3]. In addition, it was also found that EXOs of PD patients could induce dopaminergic neurons degeneration and motor deficits when injected into the striatum of mice [46,51,52,53].

Both in vitro and in vivo testing had demonstrated that microglia could internalize the α-syn-containing EXOs, inducing microglia activation and α-syn aggregation [51]. The stimulation of BV-2 microglia via α-syn could lead to the release of EXOs, which could induce apoptosis of recipient neurons. In addition, the accumulation of α-syn after microglial activation could lead to motor deficits resulting from losing dopaminergic neurons [45,51,52].

It was indicated that both small and large structures of α-syn could travel within EXOs among neurons, which resulted in neurotoxicity. This was proved by the over-expression of α-syn in SH-SY5Y neuroblastoma cells that led to its exosomal release [41]. In addition, both monomeric and oligomeric α-syn were detected inside the EXOs [41,46,47,54].

It was proven that the autophagy–lysosomal pathway was also involved in α-syn transmission through EXOs. This could be observed in cases of lysosomal inhibition in donor cells as it was found to increase exosomal release and transmission of α-syn [41,42]. The MVB and amphisomes effects are promoted as a compensatory mechanism to prevent excessive aggregation of intracellular α-syn. Although this mechanism initially decreases the intracellular α-syn levels, which seems a protective measure, it leads to the enhanced transmission of α-syn through exosomal release, resulting in PD progression and an over-loaded extracellular space with neurotoxic α-syn [41,55,56].

Microglia’s principal function is to degrade extracellular α-syn and remove aggregated proteins in different brain areas through phagocytosis. The microglia-mediated neuroinflammation was provoked by α-syn inducing the release of pro-inflammatory cytokines, which were proved to be involved in PD progression and pathogenesis [57,58]. The exact mechanism was illustrated that α-syn stimulates microglia via the Kv1.3 voltage-gated potassium channel, leading to a significant increase in neuroinflammation and neurodegeneration [59]. Furthermore, activated microglia-releasing EXOs are responsible for transmitting inflammatory mediators, such as IL-1β, IL-18, and TNF-α, from glia-to-glia or glia-to-neuron, which are the most common vital reasons for induction of dopaminergic neuron degeneration in PD patients [57,58]. Then, this vicious cycle continues as those neurons release α-syn-containing EXOs that activate microglia, repeating the whole process.

## 4. EXOs as a Future Approach for PD Diagnosis

The current approach for PD diagnosis is based on neurological symptoms and imaging techniques for the brain. Those methods are usually imprecise and time consuming, requiring the physician to monitor the patient for some time before confirming the diagnosis [60,61,62]. Biomarkers with a high level of reliability are necessary for the future of PD diagnosis, disease progression monitoring, and treatment response assessment [61,63]. The most challenging obstacle in identifying reliable biomarkers is that PD is restricted in a certain subset of neurons in a specific location of the CNS. Based on this view, EVs released from those specific neural cells circulate in CSF, and blood can be PD biomarkers as indicated in Table 1 [64].

Various cells in the CNS, such as microglia and astrocytes, can release EXOs, which are initially thought to be a mechanism by which the cell eliminates unnecessary proteins. However, recent work suggested a pivotal role for these organelles as mediators for cell-to-cell communication. As discussed before in previous sections, EXOs are the potential culprit in the PD progression [54,65]. One reason that makes it crucial to study the involvement of EXOs as diagnostic biomarkers in PD is its consistent relationship with various pathological mechanisms in PD. EXOs can help dispose of damaged organelles by generating mitochondrial-derived vesicles and thus participate in mitochondrial quality control (MQC). Abnormal EXOs secretion and MQC breakdown are both alleged in the pathogenesis of PD by triggering the disposition of α-syn and consequently leading to dopaminergic neurotoxicity [66]. Another mechanism is crucially linked to the impaired EXOs release in sporadic and hereditary PD patients [67]. The autophagy–lysosome pathway (ALP) regulates EXOs production and release by autophagy induction which promotes EXOs release, and autophagy stimulation which inhibits EXOs release [68,69]. This impairment indicates that EXOs release may increase PD [67]. Indeed, ALP modulators as bafilomycin can prevent autophagosome–lysosome fusion, increasing α-syn release from EXOs [42,55]. Smaller oligomers were released via EXOs and RAB11A-associated pathways, while high-aggregated gamma-syn was secreted by membrane shedding [55].

The environmental contaminant rotenone (RTN) inhibits mitochondrial complex I, causing PD [70]. RTN inhibits mitochondrial, autophagic, and lysosomal processes [71]. Enteric neurons treated with RTN produce more EXOs-containing α-syn [72]. On the other hand, rapamycin, an autophagy enhancer, unlike the RTN, reduced the α-syn oligomer released from EXOs. Collectively, it can be assumed that increasing autophagic activity reduces EXOs release, thus protecting neurons from neurological damages [73].

Moreover, EXOs contain different proteins and miRNAs that play critical roles in the occurrence of PD. For example, after analyzing the genomic sequences of miRNA-133 obtained from PD patients’ blood samples shows an increase in the frequency of miR-133b variant (90 del A) in PD patients. The latter is involved in dopamine neuron survival in mice [74,75]. In the upcoming sections, we have analyzed the results obtained from different delivery methods for miRNA-based therapy using exosomes as a core-shell to potentiate neuronal survival and differentiation. Thus, EXOs are considered a unique class of PD biomarkers, with less or no invasion [61,65,76,77]. Collectively, the previous data implies that the study of substances packaged in body fluids and released by EVs may help us comprehend the connection between systemic PD inflammation and its potential incrimination as a biomarker for PD.

Jiang et al. studied protein changes in serum EXOs derived from severe or moderate PD patients, and 14 proteins were significantly changed [78]. PD patients showed high expression of seven proteins, including pigmented factor produced from epithelium, afamin, and apolipoprotein D and J. Seven proteins had decreased expression in PD patients, including C1q supplements IGLV1-33 clusters.

miRNAs are enriched in EXO-like vesicles secreted from different types of cells, including neural cells involved in the progression of PD [61]. Those vesicles were found in different sizes ranging from 40–100 nm, assessed in CSF, blood, saliva, urine, and tears. miRNAs have shown their potential in the prognosis and determination of staging and development of PD [61,79]. As prognostic biomarkers, they can distinguish between different stages of the disease, which is ultimately crucial in the treatment and diagnosis of PD patients. Gui et al. studied reliable biomarkers, using a list of 24 miRNAs showing significant differences between patients with PD. Healthy controls showed specific results indicating that miR-1, miR-19b-3p, miR-153, miR-409-3p, miR10a-5p, and let-7g-3p are reliable biomarkers of PD. Those miRNAs in CSF demonstrated sufficient specificity and sensitivity to discriminate between PD and Alzheimer’s disease (AD) patients [61,80,81,82,83,84,85,86].

The stability of EXOs containing miRNA had been studied and assured for being utilized as biomarkers for PD. Compared with control groups, using RT-qPCR analysis, it was found that miR-24 and miR19b were significantly downregulated in patients with PD, whereas miR-195 was upregulated, representing high reliability and reproducibility as biomarkers [61,87]. Therefore, the level of miRNAs such as miR-195, miR-19b, and miR-24 can be correlated with PD progression. Other types of miRNA have the potentiality to be used as effective and dependable biomarkers, but some related information cannot be demonstrated due to different limitations toward sample size and test reproducibility [61].

Recent studies found that EXOs secretion was remarkably higher in PD patients than in healthy controls attributed to the primary role EXOs in transferring α-syn, which promotes their production to transfer the aggregated α-syn [52,65,88]. From in vivo experiments, pathological α-syn containing EXOs could be taken up by healthy neurons and induce a similar cascade of pathological events as seen in the PD brain. Thus, an efficient experiment that illustrated the importance of EXOs as a diagnostic biomarker could be efficiently designed and performed on this basis [65,86]. Microglial α-syn-containing EXOs were inoculated, leading to the induction of α-syn aggregation, causing the development in PD in mice. In addition, these EXOs have triggered the loss of dopaminergic neurons leading to PD-like motor deficits with time [65,86]. Another protein associated with an increased risk of PD is LRRK-2, a kinase enzyme encoded by the LRRK2 gene in humans, which is a member of the leucine-rich repeat kinase family. Mutations in LRRK-2 can enhance the auto-phosphorylated LRRK-2 protein levels, and they are the most commonly known cause of inherited PD. The LRRK-2 protein includes two enzymatic domains proven to have guanosine triphosphatase (GTPase) and protein kinase activity. One of the most abundant autophosphorylation sites is in the Serine-1292 residue near the GTPase domain. This autophosphorylation at Serine-1292 (pS_1292_) leads to neurotoxicity since these proteins can be packaged into EXOs. Thereby, measuring the EXOs-harbored LRRK-2 may be of particular importance as a biomarker in PD diagnosis. Fraser et al. found elevated pS_1292_- LRRK-2 levels in urinary EXOs from mutation carriers versus controls [89].

Moreover, a discrete difference between PD patients’ saliva components and those obtained from healthy controls was observed. The proteomic study by Figura et al. showed that the saliva from 39 PD patients had lower concentrations of S100-A16, ARP2/3, and VPS4B than the control group [90]. These proteins are involved in various metabolic and inflammatory pathways. The S100A 16 is a protein that potentially participates in calcium-binding and adipose tissue formation [91]. Several studies confirmed the association of the S100 protein family with PD [92] and other neurological diseases such as AD and neuronal injury [93,94,95]. The actin-related protein 2/3 complex (ARP2/3) protein is involved in forming the actin network in the cytosol, thus participating in cell motility [96]. As for the VPS4B, it was observed to decrease in PD patients’ saliva. VPS4B is involved in the endosomal vesicular release of a subset of proteins and the EXOs secretion pathway [97]. The authors suggested that the observed decrease in VPS4B might be due to its increased uptake by EXOs [90].

Furthermore, Galindez et al. [98] revealed elevated heme oxygenase-1 (HO-1) concentrations in the PD group compared to controls. Heme oxygenase (HMOX) is an enzyme that degrades heme into biliverdin, ferrous iron, and carbon monoxide [99]. In response to oxidative stress, the isoform (HO-1) becomes a culprit contributing to a more permeable BBB and the marked increase in iron deposition in the PD brain [100]. Of note, the HO-1 protein in human biofluids, including saliva, is localized mainly in EV compartments such as EXOs [101]; thus, future studies could focus on evaluating the level of EXO content of HO-1 protein in PD patients.

**Table 1:** Exosomal biomarkers isolated in cohort studies for PD diagnosis.

Source	Potential Biomarkers	Findings	*p*-Value	Patient N	Ref
Plasma	CNS-derived EXOs α-syn	↑	*p* = 0.004	267 PD, 215 controls	[88]
	miR-331-5p	↑	*p* < 0.05	52 PD, 48 controls	[102]
	miR-505	↓			
Serum	Pigmented epithelium-derived factor, Afamin, apolipoprotein D, and J	↑	*p* < 0.05	20 PD, 10 controls	[78]
	Complement C1q	↓			
	miR-19b	↓	*p* < 0.05	109 PD, 40 controls	[61]
	miR-24, miR-195	↑			
CSF	α-syn	↓	*p* < 0.05	76 PD, 58 controls	[103]
	miR-1 and miR-19b-3p	↓	*p* < 0.05	47 PD, 27 controls	[86]
	miR-153, miR-409-3p, miR-10a-5p, and let-7g-3p	↑			
Urine	SerP-1292 LRRK2/total LRRK2 ratio	↑	*p* = 0.0014	79 PD, 79 controls	[89]
Saliva	S100-A16, ARP2/3, and VPS4B	↓	*p* < 0.05	24 PD, 15 controls	[89]

## 5. Treatment of PD

Treatment with L-Dopa represents a key pillar for relieving PD symptoms [104], and it is associated with significant improvement in motor functions assessed by the Unified Parkinson’s Disease Rating Scale (UPDRS), compared to other drugs [105,106,107,108]. In addition, the available drugs to treat PD suffer from pharmaceutical limitations, including low bioavailability, extensive metabolism, short tt2, and peripheral side effects. Treatment options for PD along with their challenging pharmacokinetic and peripheral side effects are discussed in Table 2.

### 5.1. Challenges of PD Treatment

Several limitations were observed when using L-Dopa for a long-term duration. For example, after only two years of exposure to L-Dopa, one-third of the patient described the development of various types of motor response oscillations and drug-induced dyskinesias, which may impede the benefits after initial treatment [144]. Earlier in 1990, in vitro studies demonstrated that high doses of L-Dopa can induce toxicity in dopaminergic neurons [145,146,147], which led some specialists to withdraw the recommendation for this drug [148]. However, pathological reports and autopsies from long-term exposed patients did not show evidence for substantia nigra degeneration [149,150,151].

L-Dopa’s pharmacological and physiological properties increase the difficulty of its delivery [152]. Meanwhile, the main obstacle for optimizing the delivery of L-Dopa to the brain is the pathway the prodrug will have to take to pass the BBB efficiently, starting from its absorption site in the gastrointestinal tract to its conversion via aromatic decarboxylase and catechol-*O*-methyl transferase (COMT). These effects reduce L-Dopa biological tt2 and consequently it will lead to an insufficient delivery to the brain in addition to the conceived competition at the transport site to enter the brain [153]. Therefore, high doses of L-Dopa are required for an effective treatment which will be an object of debate since L-Dopa was not subjected to toxicological testing [152].

L-Dopa absorption from the gut is a subject of various limitations. First, L-Dopa is absorbed in the intestine and can be affected by the slow gastric emptying rate (GER) or constipation, which are the non-motor symptoms of PD [154]. Furthermore, delayed GER will reduce the bioavailability by inducing bacterial overgrowth, which may affect the rate at which L-Dopa reaches its absorption site. Second, since the chemical structure of L-Dopa is an alanine derivative, the amino acids from the diet interfere with the absorption of L-Dopa, hindering its absorption [155,156,157,158]. Third, it is estimated that only 1% of the administered drug will make it to the brain due to the ubiquitous distribution of DOPA decarboxylase that is spread in almost every tissue of the human body so that the dopamine will be degraded in multiple unwanted sites. Thus a significant proportion of the drug will no longer be available to pass the BBB [159]. Finally, in the last step in the delivery, a competition at the amino acids transporters will impede L-Dopa delivery to the substantia nigra [157].

### 5.2. Therapeutic Aspects of EXOs in PD

**Figure 4:**
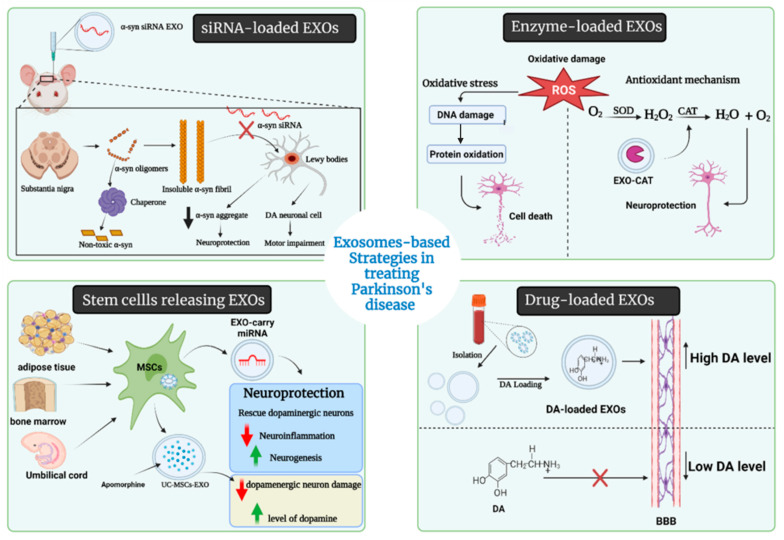
Therapeutic approaches of EXOs in PD.

The blood–brain barrier (BBB) is a highly selective permeable membrane that thoroughly limits the transportation of large molecules and almost all tiny molecules from other organs to the brain. For this reason, various invasive techniques have been developed or under development to overwhelm the selectivity of the BBB, such as neurosurgery, the biochemical opening of the BBB, and different formulations of nanoparticles [160,161,162]. However, those techniques also have problems in terms of drug delivery, such as rapid drug clearance by the mononuclear phagocyte system (MPS). Several reports indicate that EXOs can cross the BBB and overcome the immune-special status to reduce drug clearance by MPS. More importantly, EXOs can spread and transport proteins and RNAs into the brain through intranasal, intravenous, intraperitoneal, and intracranial administration, which indicates the high flexibility and compatibility of EXOs-based drug delivery in treating CNS diseases [162,163,164]. Several drugs had been enveloped into EXOs for delivery uses in the past decade. Alvarez-Erviti et al. 2011 reported the delivery of small interfering RNAs (siRNAs) through EXOs into the mouse brain by tail vein injection [162]. Different therapeutic approaches are presented in Figure 4, detailed in Table 3, and discussed as follows.

#### 5.2.1. Drug-Loaded EXOs

To date, there are no curative approaches that halt the course of PD. Only the treatment that temporarily replaces the missing neurotransmitters is currently available. Most of what is known as a difficulty for current PD treatments resides in the inability to cross the BBB. The drug can be loaded to EXOs to curtail these concerns, based on their ability to mediate the neuronal communication in the CNS and pass through the BBB [172]. EXOs could be harnessed to deliver CNS-acting drugs to treat and alleviate PD symptoms since they can adhere to the specified cell surface and release their cargoes [29,173,174,175].

As mentioned previously, PD occurs when the dopaminergic neurons in the substantia nigra pars compacta have been damaged, and consequently, the DA level decreases [176]. Therefore, DA administration to the brain to compensate for the reduction in DA level is proposed, but the limitation is to cross the BBB in the therapeutic dose. Thus, L-Dopa shows a promising therapeutic action because it is converted into the parent drug in the brain [177].

Even though L-Dopa can reach the brain, delivering a sufficient amount to the targeted regions is challenging. Unfortunately, a small amount of L-Dopa can pass to the brain due to the destruction of L-Dopa in plasma by the decarboxylase enzyme [178]. Thus, researchers interest increase in drug delivery systems such as ligand-modified nanoparticles [179], micelles [180], mesoporous nanoparticles [181], dendrimers [182], and EXOs [169]. These systems can allow the direct use of DA in the brain. Compared to EXOs, ligand-modified nanoparticles, micelles, and dendrimers show modest results to pass through the BBB [182]. However, EXOs have many ways to override this obstacle, and they can cross the BBB and reach the drug to its target. The proposed underlying mechanism is that EXOs can cross through the endothelial cells by “Jumping”, undergo transcytosis, and eventually release their content [183]. Another suggested mechanism is that EXOs can break down the vascular endothelial barrier in the BBB to enhance the penetration [184].

Moreover, transferrin receptor (TFR) is highly expressed by brain capillary endothelial cells forming the BBB and is therefore considered a potential target for brain drug delivery [185]. Based on the transferrin–TFR interaction, EXOs can bind to the transferring receptors and get to the brain. Qu et al. collected blood EXOs from a mice model. Then, they encapsulated DA into these vesicles, and the obtained formulation was systematically administered to the mouse model of PD [169]. The results showed a significant decrease in the amphetamine-induced rotation test compared to the control group. This test tool is used to predict the extent of motor impairment in PD [186]. In addition, the amount of DA in lesioned striatum significantly increased compared with the control [169]. The used EXOs in this experiment were blood derived, specifically from reticulocytes. The use of blood EXOs as drug carriers with appropriate loading efficacy is considered a potential future candidate to treat PD.

Additionally, quercetin, a plant flavonol from the flavonoid group, is considered a supplemental therapy for PD [187]. However, this flavonoid has poor brain targeting activity and low bioavailability, limiting its effect in lesioned brain areas [188]. Quercetin loading into EXOs (EXO-Que) showed better results in other NDDs, including AD, which dictates future research on flavonoid-loaded EXOs in PD.

#### 5.2.2. Enzyme-Loaded EXOs

The unmet medical need for PD treatment is undoubtedly known for every medical practitioner. To solve this dilemma, scientists used enzymes that might interfere in the pathological cascade for PD, such as catalase (CAT), labeled it to EXOs, and considerable results were obtained. CAT-loaded EXOs is a novel therapeutic approach that has been studied by Haney et al. subjected to in vitro and in vivo studies [163]. CAT is a large protein referred to as a major antioxidant enzyme, and it neutralizes the deleterious effects of reactive oxygen species (ROS), which is the major component in the cascade of PD pathological mechanisms [189].

Throughout Haney et al.’s experiments, CAT was the payload of EXOs and was incorporated into these vesicles using different methods to test which one was most suitable as a drug loading technique. They used sonication, incubation at room temperature (RT), freeze/thaw cycles, or extrusion procedures. The obtained EXO-CAT formulations using sonication and extrusion showed the highest catalytic activity, followed by those obtained by freeze/thaw cycles and then the incubation at RT. Interestingly, the results were optimistic and proved the extraordinary ability of these tiny vesicles to reach the CNS, interact with the targeted neurons and deliver the incorporated CAT. Furthermore, CAT molecules showed an efficient deactivating effect on ROS; thus, it provided a prompt neuroprotective ability in PD. The experiment by Haney et al. showed the most promising results after sonication or extrusion [163].

#### 5.2.3. EXOs-Loaded Short Hairpin (sh-), si-, and miRNA Molecules

The degeneration of nigrostriatal dopaminergic neurons leads to the accumulation of intracellular molecules known as Lewy bodies, which are composed of a spherical matter called α-syn [7,190]. Thus, decreasing α-syn expression through gene knockdown may be a responsible approach for treating PD. Silencing α-syn transcription by siRNA exhibits a priority in the field of gene therapy by a phenomenon referred to as RNA interference (RNAi) [191].

A study by Lewis et al. reported that infusion of modified siRNA against α-syn into the hippocampus of normal mice downregulated the α-syn over 14 days [192]. Although promising results were documented on the application of siRNAs, their effect on cerebral regions was hampered because of the difficulty of targeting specific tissues or cell types, immunogenicity, and a short-term gene downregulation lasting for 3–7 days after injection [167].

An untapped effective siRNA delivery might be provided using natural core-shell EXOs because they could cross the BBB [164].

From the genetic perspective, siRNA-loaded rabies virus glycoprotein-EXOs (RVG-EXOs) were reported as a potential silencer for α-syn mRNA expression in the substantia nigra, exhibiting a significant effect in treating PD. The siRNA-loaded EXOs showed less efficacy in reducing ROS levels in PD-derived tissues than curcumin (CUR) since CUR-loaded EXOs had neuroprotective properties and could readily decrease the α-syn aggregates [193]. For this reason, a combination of both treatments might synergistically reduce the α-syn cytotoxicity on the nigrostriatal dopaminergic neurons.

Other non-coding RNA had been loaded to EXOs and showed modulatory disease attenuation effects [162,166,194,195]. Utilizing shRNA carried on adeno-associated virus inhibited the expression of endogenous α-syn [194] when loaded to which were double-stranded DNA vectors (minicircles; MCs). This exosomal platform expanded the gene silencing period to over seven weeks, which was longer, making it applicable for chronic PD cases and reducing the injection frequency and cost for patient satisfaction [168]. Izco et al. extracted the RVG-EXOs from primary dendritic cells and loaded them with shRNA-MCs [168]. The in vivo results were quite promising, with a potential decrease in α-syn protein levels over a prolonged period. However, the systemic injection into a mouse failed, as described by the results after 30 days of follow-up. The results showed no significant changes in the α-syn mRNA levels in the ipsilateral or contralateral brain regions and showed an observed significant increase in the α-syn protein levels in the striatum. Nevertheless, after 90 days, positive responses in the substantia nigra pars compacta, and other brain regions were observed, which reflected the potential action of the RVG-EXOs delivered shRNA-MCs on a longer duration.

The pathological hallmarks of PD correlate with miRNA’s expression level, which is abundantly expressed in the CSF of PD patients and constitutes a protective environment for genetic materials. EXOs-based miRNA delivery exhibits the possibility to alter BBB integrity. Thereafter, the administration of EXOs with a high abundance of specific miRNAs may become a next-generation miRNA-based therapy that develops from the conventional direct application of miRNAs as therapeutic drugs. Currently, two different methods have been embraced to explore the potential therapeutic application of exosomal miRNAs as drugs to treat NDDs. The first one is to administrate EXOs containing valuable therapeutic miRNAs directly. At the same time, an alternative approach for in vivo studies is to package selected miRNAs that eliminate disease-associated genes or improve neurodegenerative factors into EXOs [162]. In the case of PD, treatment with miR-188-3p-enriched EXOs can suppress autophagy and pyroptosis by targeting the core molecules of inflammasome CDK5 and NLRP3 [166]. miR-7 is another miRNA whose expression allows normal development and neurogenesis in the CNS and keeps α-syn at the physiological level [195]. Moreover, miR-30a-5p is a factor in the pathogenesis of PD by regulating ubiquitin-mediated degradation of glutamate transporter 1, and it ameliorates the motor deficits and the pathological consequences [196]. Thereby, loading these molecules into EXOs is a nuanced approach, though it is still in its infancy since it harbors several beneficial effects and deserves to be studied in future experimental designs and clinical trials [197].

#### 5.2.4. EXOs as Nanoscavengers

A biological nansoscavenger is a molecule that targets toxins in specific regions and induces a deterrent effect. From this concept, EXOs can be regarded as nanoscavengers carrying different cargos such as RNA, and miRNA drugs such as quercetin and CUR, and even enzymes such as neprilysin and insulin-degrading enzyme. EXOs succeed in drawing an in-depth look for their future usage in clinical studies for AD [198]. Similarly, nanoscavenger in PD may pave the way for future treatment choices.

More recently, Liu et al. designed a core-shell hybrid system called a nanoscavenger by immature dendritic cells (imDC)-derived EXOs loaded with hydrophobic CUR and hydrophilic siRNA molecules. These molecules were combined into one shell using the phenylboronic acid-poly(2-(dimethylamino) ethyl acrylate) nanoparticle. The nanoscavenger effectively delivered the gene-chem drug to the targeted brain regions [165].

After the tenth administration, the results showed a significant slow movement speed and reduced the time needed to tip the rod in the treated mice. Moreover, an immuno-suppressive effect was demonstrated after nanoscavenger treatment. Furthermore, an increase in Fox p3 in CD4^+^ T cells and a decrease in the IL-22 and IL-17 cytokines were observed. In addition to the reported efficacy, the safety of this treatment was tested in the mice model, and no toxicity or induced organ damage was revealed. The results from the experiment are promising, knowing that it is the first study to combine CUR with the component siRNA into one system using the core EXOs to target α-syn.

### 5.3. Stem Cells-Derived EXOs

Mesenchymal stem cells (MSCs) are multipotent progenitor cells that can be isolated from several tissues, including bone marrow (BM), adipose tissue, umbilical cord (UC), and placenta, because of their anti-inflammatory, anti-apoptotic and immunomodulatory properties. MSCs have been used in clinical trials for various disorders, including NDDs [199,200]. In addition, EXOs derived from MSCs have been considered effective for treating various pathological conditions, including CNS disorder, and their ability to rescue dopaminergic neurons in neurotoxic synthetic organic compound 6-hydroxydopamine (6-OHDA) mice models of PD. Furthermore, MSCs-derived EXOs provide a potential PD treatment since they carry beneficial miRNAs and interact with neuronal cells to decrease neuroinflammation and stimulate neurogenesis in PD animal models [201].

EXOs isolated from various cells can be manipulated to target specific neurons and brain regions, making them potentially therapeutic for PD and other NDDs [202]. For example, EXOs derived from UC-MSCs enriched apomorphine-produced asymmetric rotation by lowering the damage of dopaminergic neurons in the substantia nigra and enhancing DA levels in the striatum [203].

## 6. Conclusions

As a nano-vesicular system, EXOs could be vehicles for different molecules, including proteins, nucleic acids, miRNAs, and even drugs; therefore, they attracted great interest as diagnostic and therapeutic players in PD. These nano-vesicles possess a role in PD propagation by spreading misfolded α-syn through a cell-to-cell communication-based mechanism. Compared to other drug delivery systems, the uniqueness of EXOs lies in their ability to penetrate the BBB and the low immunogenicity of the natural vesicles derived from cell membranes. Consequently, a future outlook to applying EXOs in delivering various cargos, including miRNAs, shRNA, siRNAs, enzymes, and drugs, is an intriguing approach. Isolated EXOs from PD patients have led to identifying novel biomarkers for PD, a promising prospective diagnostic and prognostic tool for this disease. However, EXOs were detrimental in PD patients; their pros can be exploited to treat NDDs.

## Figures and Tables

**Table 2:** The challenging pharmacokinetic and peripheral side effects of PD treatment options.

Name/Chemical Structure	Class	Pharmacokinetic Features	Peripheral Side Effects	Ref
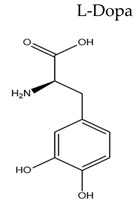	Dopamine Precursor	Oral Fa (%) = 31 to 33% for 1g/oral dose Short tt2 ≈ 30 to 60 min for oral LDopa	Upset GIT Cardiac arrhythmia Hypertension “on/off” phenomena. Dyskinesia on long-term therapy (75%) Postmenopausal bleeding	[109,110,111,112,113,114,115]
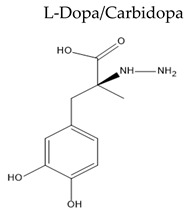	Carbidopa is a peripheral DOPA decarboxylase inhibitor	Oral Fr(%) for LDopa for controlled release formulation = 40–70%, and ≈ 58% for carbidopa Short tt2 ≈ 1.3 h	Similar to those of LDopa but for a lesser extent. On/off phenomena is reduced since the pulsatile dosing manner, and consequently the fluctuation in absorption and metabolism decreased	[116,117]
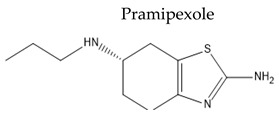	Non-ergoline dopamine agonists	Oral Fa (%) > 90% tt2 = 8–12 h Minimal metabolism	Orthostatic hypotension Constipation Peripheral edema Urinary frequency Visual abnormalities	[118,119]
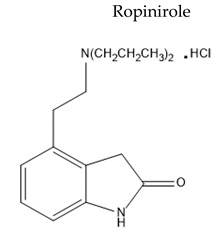		Oral Fa (%) ≈ 50 tt2≈ 6 h Extensivly metabolized by CYP1A2	Orthostatic hypotension Peripheral edema Somnolence Dyskinesias	[120,121,122]
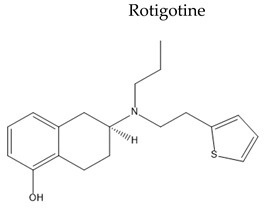	Non-selective non-ergoline dopamine agonist	Poor oral bioavailability Transdermal Fa (%) ≈ 37% tt2≈ 5–7 h Extinsively metabolized by glucuronidation	Nausea Application site reactions Vomiting Fatigue	[123]
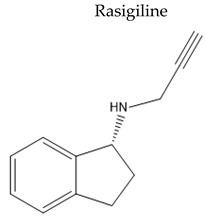	MAO B inhibitors	Oral Fa (%) ≈ 36 Short tt2 ≈ 1.34%	Anorexia and weight loss Orthostatic hypotension	[124,125]
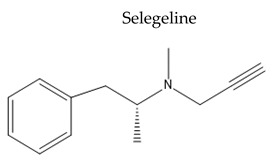		Oral Fa (%) ≈ 4.4% Transdermal Fa (%) ≈ 73% tt2 ranged from 15–25 after IV and transdermal delivery and 9–15 for oral dosing	Constipation Insomnia Application-site reactions for transdermal delivery Peak dose dyskinesia	[126,127]
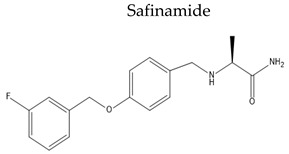		Oral Fa (%) ≈ 95% tt2 = 20–30	Dyskinesia Retinopathy Backache Constipation	[128]
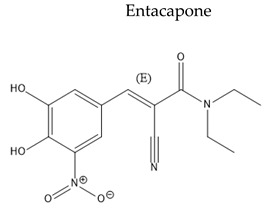	COMT inhibitors	Oral Fa (%) ≈ 25–35% Extensivly metabolised tt2 = 2.40 ± 1.70 h	Urine discoloration Diarrhea Peak dose dyskinesia Gastrointestinal effects	[129,130]
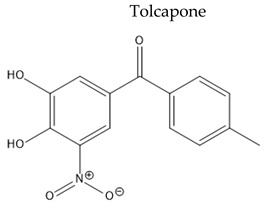		Oral Fa (%) ≈ 65% Short tt2 = 2–3 h Extensively metabolized by COMT	Abdominal pain, diarrhea Dyskinesia Increase liver enzyme (fulminant hepatitis)	[131,132,133]
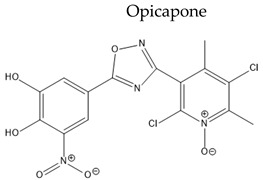		Oral Fa (%) ≈ N/A Short tt2 = 0.8 h in small doses (50 mg)	Dyskinesia Constipation Dry mouth Insomnia	[134,135]
** 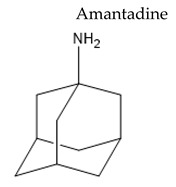 **	Weak dopamine agonist with some antimuscarinic activity and N-methyl-D-aspartate antagonist	Complete oral bioavailability in healty young subjects tt2 ≈ 14.3 (l0.2 to 31.4) h Oral Fa (%) = 40–60% in horses with tt2≈ 3.4 ± 1.4 h	Dry mouth Constipation Orthostatic hypotension Syncope or falls Peripheral edema Urine retention	[136,137,138,139]
** 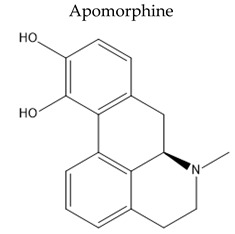 **	non-ergoline dopamine D1 and D2 agonist	Sublingual Fr (%) = 17–18% relative to SC Short tt2 ≈ 45 min About 60% of the sublingual dose is eliminated as a sulfate conjugate	Constipation Sweating, Salivation Orthostatic hypotension	[140]
** 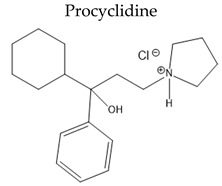 **	Antimuscarinic anticholinergic drugs	Oral Fa (%) ≈ 75% tt2 is up to 12 h Metabolized 20% by liver CYP 450	Constipation Urinary retention Blurred vision Tachyarrhythmia	[141,142]
** 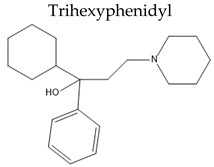 **		Oral Fa (%) ≈ 100% tt2= 10.2 ± 4.7 h	Dry mouth Blurred vision Abdominal discomfort Constipation Tachycardia Urinary retention	[114,143]

L-Dopa: Levodopa, Oral Fa (%) absolute oral bioavailability, oral Fr (%) relative oral bioavailability, transdermal Fa (%) absolute transdermal bioavailability, tt2: elimination half-life, DOPA decarboxylase: Aromatic L-amino acid decarboxylase, (COMT) catechol-*O*-methyl transferase enzyme, MAO B, monoamine oxidase enzyme, SC: subcutaneous, N/A: not applicable.

**Table 3:** Applications of exosomes in PD treatment.

Cargo	Vesicle Size (nm)	Source	Isolation Method	Loading Method	Therapeutic Efficacy	Ref
CAT	100–200	Mouse macrophage	Differential centrifugation followed by filtration	Incubation with or without saponin, freeze-thaw cycle, sonication, or extrusion	Enhanced CAT bioavailability in neuronal cells, therefore, increased therapeutic efficacy and decreased ROS level in the brain	[163]
CUR and siRNA molecules	70	imDC	Differential centrifugation followed by ultrafiltration and passed through a size exclusion chromatography	Sonication	Observed slowness in movement speed, an improvement in the time to tip of the rod and an immune suppressive effect, an increase in Fox p3 in CD4^+^ T cells and a decrease in the IL-22 and IL-17 cytokines	[165]
miR-188-3p	-	ADSC	Differential centrifugation	Culturing cells with miR-188-3p-overexpressed EXOs	Alleviated the damaged substantia nigra and suppressed the levels of CDK5 and NLRP3 in the PD mice model	[166]
siRNA	-	BMDCs		Electroporation	A significant decrease in total α-syn mRNA and protein level	[167]
shRNA-MCs	-	DCs transfected with RVG-Lamp2b.			Reduction in the α-syn aggregation and loss of dopaminergic neurons	[168]
L-Dopa	40–200	Blood of Kunming mice		Incubation	Boosting the brain delivery of DA	[169]
DNA aptamers	100	myc-RVG-lamp2b			PFF-induced insoluble α-syn aggregates were reduced, therefore reducing PD progression	[170]
GDNF	96.0 ± 9.1	Macrophages			Enabled GDNF to reach CNS and consequently induced a neuroprotective effect, and reduced inflammation and levels of activated microglia in the targeted regions	[171]

BMDCs: Murine dendritic cells from bone marrow; ImDC: immature dendritic cell; ADSC: Adipose-derived stem cell; DCs: Primary dendritic cells; RVG: Rabies virus glycoprotein; Lamp2b: lysosomal associated membrane protein-2; Myc-RVG-lamp2b: Mice rabies virus glycoprotein-lysosomal-associated membrane protein-2; PFF: performed fibrils; GDNF: Glial cell line-derived neurotrophic factor.

## Data Availability

No new data were created or analyzed in this study. Data sharing is not applicable to this article.
